# Research Note: Preliminary results, first detection of *Enterococcus cecorum* from environmental samples by streaking on X-Gluc containing selective media

**DOI:** 10.1016/j.psj.2023.103253

**Published:** 2023-10-31

**Authors:** Jesper Tessin, Judith Rohde, Arne Jung, Nicole Kemper, Jochen Schulz

**Affiliations:** ⁎ Institute for Animal Hygiene, Animal Welfare and Farm Animal Behaviour, University of Veterinary Medicine Hannover, 30173 Hannover, Germany; † Institute for Microbiology, University of Veterinary Medicine Hannover, 30173 Hannover, Germany; ‡ Clinic for Poultry, University of Veterinary Medicine Hannover, 30559 Hannover, Germany

**Keywords:** *Enterococcus cecorum*, detection, environment, X-Gluc, direct streaking

## Abstract

The isolation of cultivable *E. cecorum* from the environment of poultry houses remains a challenge. Environmental samples (dust wipes, equipment swabs, pooled feces) and samples from culled bird vertebras were collected from an infected broiler flock on d 37 posthatching. To isolate the bacterium from the cultivable microbiota, suspensions from the environmental samples were streaked onto a blood agar base medium supplemented with 5-bromo-4-chloro-3-indolyl-beta-D-glucuronic acid cyclohexylammonium salt (**X-Gluc**), colistin sulfate, and nalidixin. The chromogenic reaction facilitated the isolation of *E. cecorum* from contaminated surfaces and pooled feces. Isolates from both the environment and vertebras were confirmed using MALDI-TOF and PCR analysis. Colony appearance and antimicrobial susceptibility tests revealed no phenotypic differences among the isolates. It remained unclear whether the isolates originated from the same clone. However, the principle of isolating the pathogen by streaking on a chromogenic agar may motivate researchers to investigate the transmission routes of infectious isolates, potentially leading to the optimization of biosecurity measures.

## INTRODUCTION

*Enterococcus cecorum* (***E. cecorum***) is an emerging avian pathogen that causes outbreaks, particularly in broiler chickens. Affected flocks exhibit lameness or hock sitting due to spondylitis and osteomyelitis, as well as increased mortality (Grund et al., 2021). Additionally, *E. cecorum* has been identified as one of the most significant antimicrobial-resistant bacteria in poultry in the European Union, posing challenges for treatment due to late etiological diagnosis and the use of antimicrobials (Nielsen et al., 2022). It is believed that *E. cecorum* can persist in the environment of broiler houses, leading to subsequent outbreaks in successive flocks. However, the transmission and sources of infections often remain unknown. Isolation of *E. cecorum* can be challenging, as its cultivation requirements are more demanding than those of other *Enterococci*, and fast-growing bacteria may outcompete *E. cecorum* (Avberšek et al., 2021). The presence of *E. cecorum* can be confirmed using molecular biological methods such as PCR, which can help identify potential sources or transmission pathways (Jung et al., 2017).

Nevertheless, the ability to cultivate and isolate the bacterium from environmental sources is essential to obtain information about infectivity, persistence, phenotypic resistance, and sufficient DNA for whole genome sequencing and typing methods. One strategy for isolating species from a microbial community involves streaking inoculums on selective media containing substrates for specific enzyme activity. In a study by Manero and Blanch (1999), it was found that, unlike other species of the genus *Enterococcus, E. cecorum* exhibits β-glucuronidase activity. The substrate 5-bromo-4-chloro-3-indoxyl-β-D-glucuronic acid sodium salt can be used to detect β-glucuronidase activity, resulting in the formation of a blue precipitate upon cleavage. Positive colonies on solid media appear blue in color (Frampton et al., 1988).

In this case study, samples were obtained from the environment of an infected flock of 37-day-old broiler chickens. A medium supplemented with antibiotics and X-Gluc was prepared as solid media for the chromogenic reaction to isolate *E. cecorum*. Presumptive isolates were identified using molecular biological methods.

## MATERIALS AND METHODS

### The Affected Flock

Samples were collected on d 37 posthatching from a forced ventilated broiler barn housing 30,020 Ross 308 birds (Aviagen, Huntsville) raised on peat moss litter. Sampling procedures conducted in the study were approved by authorities of the University of Veterinary Medicine, Hannover, Foundation (reference number AZ: TVO-2022-V-76). The birds were raised for 40 days posthatching and had been vaccinated against Newcastle disease (Avinew NeO, Boehringer Ingelheim Vetmedica GmbH, Ingelheim, Germany) on d 14, infectious bursal disease (AviPro PRECISE, Lohmann Animal Health GmbH, Cuxhaven, Germany) on d 17, and infectious bronchitis twice (Poulvac IB QX, Zoetis Deutschland GmbH, Berlin, Germany) on d 10 and (Avishield IB H120, Dechra Veterinary Products Deutschland GmbH, Aulendorf, Germany) on d 17. On d 14, the farm veterinarian observed lameness and joint (femoral head) inflammation. A treatment with amoxicillin (30 mg/kg, Dechra Veterinary Products Deutschland GmbH, Aulendorf, Germany) via drinking water started at the same day and was applied for 3 d. On d 20, the birds received amoxicillin again (30 mg/kg) and colistin sulfate (6 mg/kg, Bela-Pharm GmbH & Co. KG, Vechta, Germany) additionally until d 23. Affected broilers displayed fatigue, emaciation, and various degrees of paralysis, including resting on their hocks and caudal abdomen with both legs extended cranially. Paralyzed birds were unable to move or stand. The overall bird loss at d 40 was 3.2%, and approximately 48% (454 birds) of the deceased or euthanized broilers exhibited symptoms consistent with potential *E. cecorum* infections.

### Pooled Dust Samples

For the pooled dust sample, 18 autoclaved wipes (AlphaWipe TX1004 Dry Cleanroom Wipers, Texwipe, Kernersville) were moistened with PBS buffer and collected from 9 different locations, including the door, tools, air supplies, air exhaust, feed pans, walls, and feed lines. Two wipes were used per location and combined in a sterile Stomacher bag (Whirl-Pak Standard Bags—710 mL, Fisher Scientific SAS, Illkirch, France). All samples, including those from the barn, were transported in a cooling box to the laboratory within 3 h. In the laboratory, 20 mL of PBS buffer was added to each of the 9 pooled samples. The Stomacher bags were homogenized for 2 min at 240 rpm using a Stomacher 400 Circulator Lab Blender (Seward Ltd., Worthing, UK). The suspension from each bag was transferred to 50 mL centrifuge tubes (Tube 50 mL, 114 × 28 mm, PP Sarstedt AG & Co. KG, Nümbrecht, Germany). From each centrifuge tube, 1 mL of the suspension was pipetted into a new empty tube, resulting in a pooled sample containing 9 mL of dust suspension. Subsequently, the suspension was streaked onto the selective medium.

### Pooled Fecal Sample

A single pooled fecal sample was collected using sterile tubes equipped with sampling spoons (Feces Container 30 mL with red screw cap and spatula, VWR, Leuven, Belgium). The fecal sample was composed of feces obtained from 6 distinct locations within the barn. In the laboratory, 1 g of feces was added to 9 mL of sterile NaCl solution. The suspension was then vortexed for 30 s at 4,500 rpm before being streaked onto the selective medium.

### Samples From Carcass Buckets

Two plastic buckets (volume 10 L), which were utilized within the flock, were sampled. It is important to note that no birds were present in the buckets during the sampling process. For each bucket, a swab wetted with sterile PBS buffer (Alpha Sampling Swab TX 715, Texwipe, Kernersville) was passed once along the inner wall in a circular motion at the junction between the bottom and the wall. The 2 swabs were then combined in a single sterile centrifuge tube (Tube 50 mL, 114 × 28 mm, PP Sarstedt AG & Co. KG, Nümbrecht, Germany) for transportation to the laboratory. Upon arrival, 5 mL of PBS was added to the pooled sample, and the mixture was vortexed at 4,500 rpm for 1 min before streaking onto the selective medium.

### Sample From Carcasses

On the day of environmental sampling, 2 samples were obtained from the vertebras of 2 euthanized broilers that exhibited complete paralysis, resting on their hocks with legs extended cranially and unable to move or stand. The farmer performed emergency euthanasia of the birds. A single swab (Cotton Swab 15 cm sterile, metal, 2 mm head, WDT, Garbsen, Germany) was used by farm veterinarian to sample both vertebras of the affected animals. In the sixth thoracic vertebra, both vertebras displayed a white to reddish discoloration indicative of an inflammatory mass. After sampling, the top portion of the swab was cut using sterile scissors and placed into a 1.5 mL sterile Eppendorf tube (Eppendorf, Hamburg, Germany). For further analysis at the Clinic for Poultry of the University of Veterinary Medicine Hannover, Germany, the swab was streaked onto Columbia Agar with 5% Sheep Blood (PB5008A, Fisher Scientific GmbH, Germany) and incubated for 24 h under a 5% CO_2_ atmosphere. A subculture was prepared on the same medium for PCR analysis.

### Preparation of Selective Media

Blood agar base No.2 (CM0271, Oxoid, Fisher Scientific GmbH, Germany) without blood supplement was prepared following the manufacturer's instructions. After autoclaving, the agar was cooled down to 50°C. About 75 mg 5-bromo-4-chloro-3-indolyl-beta-D-glucuronic acid cyclohexylammonium salt (**X-Gluc**, BIMB1021, Apollo Scientific Ltd., Cheshire, UK) were dissolved in 7.5 mL of DMSO and added to the agar (final concentration of 0.075 g/L). Additionally, 10 mg colistin sulfate (455392500, Cas-Nr. 1264-72-8, Fisher Scientific GmbH, Germany), dissolved in 1.7 mL water, and 15 mg nalidixin (Cas-No: 389-08-2, AppliChem GmbH, Darmstadt, Germany), dissolved in 0.3 mL chloroform, were incorporated into the medium at final concentrations of 0.010 g/L and 0.015 g/L, respectively, as described by Ellner et al. (1966). The agar was poured into standard Petri dishes with a diameter of 90 mm. The pH of the medium was 6.9 ± 0.1 at 25°C.

### Incubation and Isolation

The prepared media were stored at 4°C for approximately 1 wk before inoculation. Media were inoculated using quadrant streaking with a sterile inoculation loop (10 µL Ps blau steril Sarstedt AG & Co. KG, Nümbrecht, Germany). As a positive control for chromogenic growth, *E. cecorum* (Strain DSM 20682T JUG) was streaked onto the plates. *E. faecalis* (Strain DSM 20478) and *E. faecium* (Strain DSM 29181) were streaked as growing controls without chromogenic reactions. The plates were incubated at 37°C in a 5% CO_2_ atmosphere for 24 to 48 h. Five single blue-green colonies which appeared similar to the positive control were selected from the samples and transferred onto fresh selective media from each medium. The plates were incubated as before. In some cases, this procedure was repeated to obtain pure cultures. Pure cultures were transferred onto TSA-Agar (CM0131 TSA, Oxoid, Fisher Scientific GmbH, Germany) and incubated at 37°C in a 5% CO_2_ atmosphere for 24 to 48 h. Suspensions from pure cultures were viewed under light microscope at 1,000× magnification. Catalase reaction was tested using a fresh 3% H_2_O_2_ solution.

### Identification of Isolates

Environmental isolates and the vertebral isolate were identified using matrix assisted laser desorption/ionization time-of-flight mass spectroscopy (**MALDI-TOF MS**) (Biotyper, Bruker Daltonik GmbH, Bremen, Germany). Sample preparation followed the manufacturer's provided protocols, employing the direct transfer method. Bacterial samples were analyzed in duplicate. Score values for identification were categorized as follows: 2.30 to 3.00 = highly probable species identification, 2.00 to 2.99 = secure genus identification and probable species identification, and 1.70 to 1.99 = probable genus identification in comparison to entries in the MBT Compass Library (revision N, April 2022).

The vertebral isolate was identified at the Clinic for Poultry, University of Veterinary Medicine Hannover, through partial sequencing of the 16S rRNA gene. Briefly, 1 colony of a pure subculture was included in the PCR reaction mix without DNA isolation. A 440-bp gene segment of the 16S rRNA was amplified using primers 91E_for (GGAATTCAAAKGAATTGAC-GGGGGC), 13B_rev (CGGGATCCCAGGCCCGGGAACGTATTCAC) and conditions according to Mignard and Flandrois (2006). PCR products were sequenced with the forward primer at Microsynth Seqlab GmbH (Göttingen, Germany). DNA sequence analysis was performed using the BLAST database of the American National Center for Biotechnology Information (Bethesda, MD).

Isolates from the environment were analyzed using a different procedure at the Institute for Animal Hygiene, Animal Welfare, and Behavior of Farm Animals, University of Veterinary Medicine Hannover. The DNA from environmental isolates was extracted using a commercial isolation kit (InnuPrep DNA Mini Kit 2.0, Analytik Jena AG, Jena, Germany), following the manufacturer's instructions. The subsequent PCR analysis was conducted using the method published by Jung et al. (2017) with modifications. The total reaction volume was reduced to 10 µL, PCR was performed for 40 cycles, and the cycle threshold value was set to 36.

### Antimicrobial Susceptibility Testing

Minimum inhibitory concentrations (**MICs**) were determined using a microbroth dilution test (M/E1-319-100 MICRONAUT-S Kleintier, Bruker Daltonics GmbH & Co. KG, Bremen, Germany) following the procedure outlined in CLSI (2023). The tested antibiotics included amoxicillin/clavulanic acid, ampicillin, cefovecin, cephalothin, chloramphenicol, clindamycin, doxycycline, enrofloxacin, erythromycin, florfenicol, gentamicin, oxacillin, penicillin G, pradofloxacin, tetracycline, and trimethoprim/sulfonamide.

## RESULTS AND DISCUSSION

**Figure 1.**
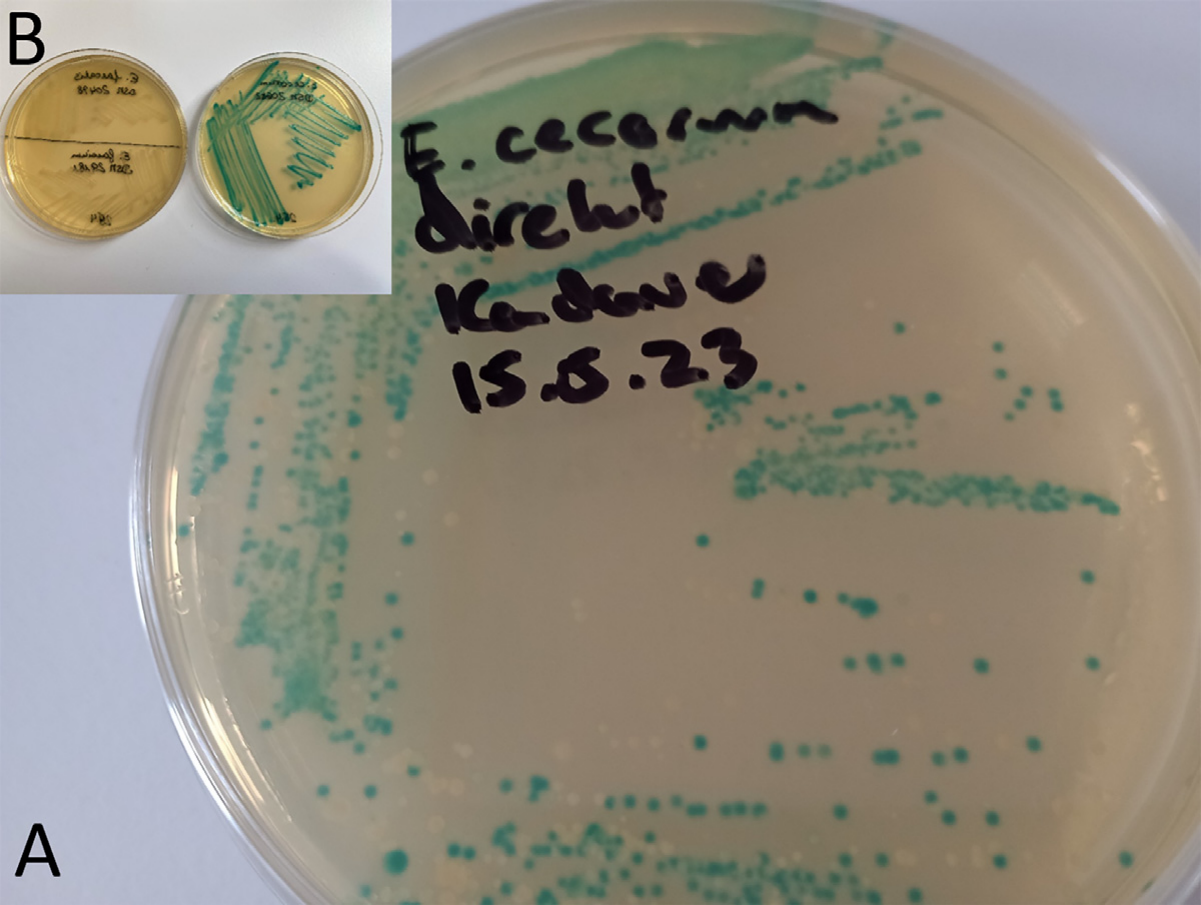
(A) Streaked suspension from a swab of carcass buckets showing stained (chromogenic) and unstained colonies. (B) Streaked chromogenic negative growing controls *E. faecalis* DSM 20478 and *E. faecium* DSM 29181 and a chromogenic positive growing control *E. cecorum* DSM 20682T JUG.

Negative controls for the chromogenic assay showed no ß-glucuronidase activity, and colonies remained colorless on the selective medium. However, when *E. cecorum* DSM 20682T JUG was cultured, it appeared as blue-green shiny colonies (Figure 1B). Streaking suspensions from environmental samples resulted in a combination of blue-green and unstained white or beige colonies (see example in Figure 1A). Out of the 15 presumed isolated colonies (5 from each environmental sample), 3 pure cultures with blue-green pigmentation were obtained from subcultures of the carcass buckets, while 1 pure culture was isolated from the pooled fecal sample. Isolating bacterial pure cultures from the pooled dust sample proved to be challenging, and the isolated colonies showed catalase-positive characteristics. Colonies obtained from the buckets and feces were highly similar to colonies from the positive control, exhibiting spherical-shaped cells under the light microscope and tested negative for catalase. Identification of these isolates as *E. cecorum* was confirmed through MALDI-TOF MS and PCR analysis. The protein spectra indicated probable species identification scores for all isolates from the barn (Table 1). Furthermore, PCR results provided definitive species identification. Susceptibility testing revealed that all isolates and the reference strain displayed similar MICs for the antimicrobials tested in the panel.

**Table 1:** Biochemical reactions, identification, and MICs of *E. cecorum* isolates and a control strain.

Isolate, origin	ß-Glucuronidase activity	Catalase negative cocci	MALDI-TOF MS species identification (score values)	PCR	Minimum inhibitory concentrations (µg/mL)
1, Thoracic vertebra	+	+	Probable (2.18, 1.81)	+	Amoxicillin/clavulanic acid 0.25/0.125–0.5/0.25 Ampicillin 0,5 Cefovecin 1 Cephalothin ≤2 Chloramphenicol 2–4 Clindamycin ≥8 Doxycycline ≥2 Enrofloxacin 0.25–0.5 Erythromycin ≥8 Florfenicol 2–4 Gentamicin 4–≥8 Oxacillin 1–2 Penicillin G ≤0.125 Pradofloxacin 0.125 Tetracycline ≥8 Trimethoprim/sulfonamid ≥4/76
2, Feces	+	+	Probable (2.09, 2.22)	+	
3, Bucket	+	+	Probable (2.11, 2.30)	+	
4, Bucket	+	+	Probable (2.08, 2.23)	+	
5, Bucket	+	+	Probable (2.23, 2.19)	+	
DSM 20682T JUG	+	+	Probable (2.34, 1.76)	+	

The isolation and identification of cultivable bacterial pathogens facilitate the analysis of phenotypic characteristics, aiding in understanding etiology and transmission. In this case, we aimed to test if β-glucuronidase activity could differentiate *E. cecorum* from other bacteria by streaking on solid media. The discovery of β-glucuronidase activity in *E. cecorum*, in contrast to 18 other *Enterococcus* spp. (Manero and Blanch, 1999), holds promise as a differentiating tool. This was further confirmed by using X-Gluc as a substrate in a solid medium, which enabled the isolation of *E. cecorum*. For example, the bacterium was isolated from a contaminated wall of a bucket, likely due to infected birds being stored in the bucket prior to sampling. The media with streaked samples from the buckets and from feces also exhibited the growth of other unstained colonies (Figure 1A, for instance), highlighting the potential for selecting the bacterium from microbial communities. However, the pooled dust sample also demonstrated β-glucuronidase activity of bacteria on the selective medium, which were catalase positive. It is well known that dust from poultry contains bacteria such as staphylococci that can grow under a CO_2_ atmosphere and possess β-glucuronidases. A more comprehensive study would likely provide additional insights into the discrimination potential of the chromogenic reaction in various environmental samples. The findings presented in this outbreak investigation may encourage scientists to evaluate the potential of a chromogenic medium in isolating *E. cecorum* from animals and environmental samples. To the best of our knowledge, this is the first report demonstrating that direct streaking of suspensions from environmental samples on a chromogenic medium enables the isolation of *E. cecorum*.

Laurentie et al. (2023) demonstrated that *E. cecorum* isolates could exhibit a wide range of MIC values and can be differentiated based on their resistances. They evaluated 23 antimicrobials using the broth microdilution method and 29 antimicrobials using the disk diffusion method. In the current study, 16 antibiotics were tested, and the MICs were identical for all isolates and antibiotics within 1 dilution step. The resistance phenotypes showed no indications that isolates from the environment and vertebrates originated from different sources. However, a more comprehensive examination of antibiotic resistances or molecular biological typing methods would be necessary to determine the phylogenetic relationship and confirm whether the isolates from the infected barn belong to the same clone. This could be explored in future studies.

We acknowledge the limitations of this case study. Nevertheless, we successfully identified *E. cecorum* from the environment by directly streaking samples onto selective media, providing clear evidence. The efficacy of these media should be further assessed in future studies. The approach of combining antibiotics and X-Gluc for discrimination holds significant potential in investigating the transmission routes of infectious *E. cecorum*. It has the potential to yield crucial information to combat this emerging avian pathogen.
